# The association between dietary diversity score and general and abdominal obesity in Iranian children and adolescents

**DOI:** 10.1186/s12902-020-00662-w

**Published:** 2020-12-11

**Authors:** Sahar Golpour-Hamedani, Nahid Rafie, Makan Pourmasoumi, Parvane Saneei, Sayyed Morteza Safavi

**Affiliations:** 1 Food Security Research Center, Isfahan University of Medical Sciences, Isfahan, Iran; 2 Department of Clinical Nutrition, School of Nutrition and Food Sciences, Isfahan University of Medical Sciences, PO Box: 81746-73461, Isfahan, Iran; 3 Gastrointestinal and Liver Diseases Research Center, Guilan University of Medical Sciences, Rasht, Iran; 4 Food Security Research Center, Department of Community Nutrition, School of Nutrition and Food Science, Isfahan University of Medical Sciences, Isfahan, Iran

**Keywords:** Dietary diversity score, Food variety, Overweight, Obesity, Abdominal obesity

## Abstract

**Background:**

To evaluate the association between diet and disease, the consideration of a whole diet has appeared to be more effective than the examination of single-nutrient intake. This study aimed to examine the relationship between dietary diversity score (DDS) and obesity in Iranian children.

**Methods:**

A cross-sectional study was conducted on 456 children aged 11–18 years, who were selected by random cluster sampling. The usual food intake for each participant assessed using a validated Food frequency questionnaire (FFQ). To calculate the dietary diversity score, food items were categorized into 5 broad groups and 23 subgroups based on the US Department of Agriculture Food Guide Pyramid. Participants were categorized based on the DDS tertile cut-off points. Anthropometric measurements were conducted based on standard protocols. Overweight and obesity were defined as 85th ≤ BMI < 95th, and ≥ 95th percentiles of BMI, respectively. Additionally, abdominal obesity was considered as WC ≥ 85th percentile.

**Results:**

Mean and standard deviation (SD) of subjects’ Body Mass Index (BMI) and waist circumference were 20.88 (SD 4.22) kg/m^2^ and 74.27 (SD 10.31) cm, respectively. The probability of overweight and obesity was increased as tertiles of DDS increased (OR among tertiles: 1.00, 1.82 and 2.13 for overweight and 1.00, 2.60 and 3.45 for obesity; this was the same for abdominal obesity: 1.00, 2.22 and 3.45, *P* <  0.001 for all). However, no statistically significant results were found after adjustment for energy intake.

**Conclusion:**

Dietary diversity positively affected obesity through higher energy intake. Despite the wide recommendation of having high dietary diversity, public health programs should emphasize to improve dietary diversity only in selective food items.

## Background

Obesity is a public health problem that affects all age groups and both genders [1]. Recently, a dramatic increase in obesity rates, especially in children, has been reported * [2]. Overweight and obesity are the emerging threats to the health status of adolescents living in both developed and developing countries * [2]. More recent data demonstrate that the worldwide prevalence of childhood overweight and obesity is approximately 25% * [3]. Iran is no exception in this respect, and approximately 15.1 and 8.3% of children are overweight and obese, respectively [4]. Children who are overweight or obese are at an increased risk of serious long-term health complications, including metabolic syndrome, insulin resistance, adulthood obesity and various forms of cancers * [5, 6]. In addition to these long-term complications, childhood obesity results in inflammation and increased inflammatory markers, related to endothelial dysfunction, cardiovascular disease and metabolic syndrome [7]. In order to reduce the financial burden of obesity complications; therefore, the prevention and treatment of childhood obesity should be considered to be public health priorities [8].

Obesity, as a complex issue, is related to individual and environmental factors [9]. Shifts in dietary patterns are a major risk factor for overweight and obesity [10]. Among dietary factors, the consumption of many nutrients, foods and food groups is associated with this health problem * [11]. Given their synergistic effects on health, and the possible interactions of food nutrients eating together [12], it is therefore better to examine indices showing the overall dietary characteristics in relation to chronic disease * [13]. The dietary diversity score (DDS), as an a priori approach of diet quality index, evaluates the nutrient adequacy and the overall diet quality [1]. A higher diversity score is associated with a better nutrient adequacy ratio and diet quality [14]. Furthermore, DDS is inversely associated with cardiovascular disease and metabolic syndrome * [15].

Although the association between individual nutrients and obesity has been widely researched, there is little evidence regarding overall dietary characteristics in relation to obesity * [16]. Most studies assessing the relation between DDS and obesity have been conducted on adults in countries other than Iran demonstrating conflicting findings * [16, 17]. Due to concerns about overweight and obesity in children, and given that few previous studies have investigated the relationship between DDS and adiposity, particularly in children; therefore, this study was conducted to determine the association between DDS, and general and abdominal obesity in Iranian children and adolescents.

## Methods

### Subjects

In this study, data were collected based on a representative sample of students aged 11–18 years who were selected by random cluster sampling method. Participants were recruited from 13 schools of 4 different districts of Isfahan city. From each school, some students were chosen randomly from the list of students in the records. Using this method, students from different socio-economic status were considered as the sample. Healthy students with no chronic disease or consuming medications that affected their food regimen or weight, including diabetes, hypertension, hypo- and hyperthyroidism, kidney diseases and liver disorders were participated in the study. Sample size was calculated in accordance with following formula with, using the information provided by previous studies [15–17]: $\left[n=\frac{z_{1-\frac{\alpha }{2}}\times {s}^2}{d^2}\right].$

After excluding participants from the study based on missing relevant information and reported energy intake outside the range of 700–4000 kcal/d, 456 out of 500 participants recruited (mean age 14 years, 189 boys and 267 Girls) were included in the study.

### Assessment of dietary intake

Dietary intake was evaluated by a self-administrated 168-item FFQ which had been assessed for validity and reliability in previous studies [18]. Frequency consumption of specified food items was reported for previous year by participants and using household measures, the portion sizes of consumed foods were converted to grams [19]. Then, consumed foods and beverages were analyzed for all energy and nutrient content using Nutritionist IV software (version 3.5.2; N-Squared Computing, Salem, OR, USA).

### Assessment of anthropometric measures

Body weight was measured using a digital scale with minimal clothing and was recorded to the nearest 100 g. Height was estimated using an upstretched tape without shoes and was registered to the nearest 0.5 cm. Using a non-stretchable tape, without any pressure applied to the surface of the body, the waist circumference (WC) at the narrowest level and hip circumference at the maximum level over light clothing were measured and recorded to the nearest 0.1 cm. Body mass index (BMI) was calculated as weight in kg divided by height in m^2^. Overweight and obesity were defined as 85th ≤ BMI < 95th, and ≥ 95th percentiles of BMI for age and sex, respectively * [20]. Abdominal obesity was considered as WC ≥ 85th percentile * [21].

### Assessment of other variables

Additional variables regarding socio-demographic information, including age, sex, parents’ educational level, household income and past medical history were obtained from a self-administrated questionnaire. The Physical Activity Questionnaire (PAQ), a self-administered, 7- day recall instrument with consistently high validity and moderate reliability [22] was used to assess the general levels of physical activity. This questionnaire containing nine items scored on a 5-point scale. A value from 1 to 5 is obtained for each item used in the physical activity composite score, and the mean of these nine items is taken, resulting in the final PAQ activity score. A score of 1 indicates low physical activity, a score of 2–4 indicates moderate physical activity, and a score of 5 indicates high physical activity.

### Dietary diversity score

Dietary diversity score was calculated based on the method using five food groups: bread/grains, vegetables, fruits, meats and their substitutions, and dairy, which was developed by Kant et al. * [23]. The main groups were divided into 23 subgroups including seven subgroups of bread/grain (refined bread, whole bread, macaroni, biscuits, corn flakes, rice and refined flour), two subgroups of fruits (fruit and fruit juice, berries and citrus), seven subgroups of vegetables (vegetables, starchy vegetables, potato, tomato, legumes, yellow vegetables and green vegetables), four subgroups of meats (red meat, poultry, fish, eggs) and three subgroups of dairy products (milk, yogurt, cheese). The participant must consume at least one-half serving of each food groups on one day to be considered as a ‘consumer’. A maximum score of 2 was awarded to each of the five groups and so that each participant received a score ranging from 0 to 10. To calculate the score of each group, the number of subgroups consumed was divided by the total number of subgroups in each main group and then it was multiplied by 2. The sum of the scores of the five main groups is reported as the total score * [24, 25].

### Statistical methods

Participants were categorized based on DDS tertile cut-off points. To compare general characteristics across DDS tertiles one-way ANOVA and chi-square test were used for continuous and categorical variables, respectively. Dietary intakes were compared using an analysis of covariance (ANCOVA). The relationship between DDS tertiles and obesity was evaluated using a multinomial and binary logistic regression, controlling for age (years) and sex in model I; physical activity (low/ medium/ high) in model II; and energy intake (kcal) in model III. In model IV, to examine abdominal obesity, BMI was further adjusted in addition to the above-mentioned variables. In all multivariate models, the first DDS tertile was considered as the reference. All statistical analysis was carried out using SPSS version 21 (SPSS Inc., Chicago, IL, USA).

## Results

The prevalence of overweight, obesity and abdominal obesity between 456 children aged 11–18 years were 17.1, 9.0, and 8.6% respectively. The mean age of study participants was 14.44 years, and the mean weight was 53.36 kg. Approximately 59% of participants were girls. The DDS mean and standard deviation were 5.93 and 0.88. The maximum and minimum diversity score was related to the fruit (1.94 ± 0.26) and meat (0.57 ± 0.28) groups, respectively. The baseline characteristics of the study participants across DDS tertile categories are shown in Table 1. Compared with participants in the lower category, those in the higher DDS category were older and had higher values in various anthropometric measurements, including weight, height, BMI and both waist and hip circumference. Participants had mostly moderate physical activity level and there were no children and adolescents with high physical activity in our sample.

**Table 1:** Baseline characteristics according to tertile of Dietary Diversity Score among children (n 456) aged 11–18 years 1

Variables		Tertile of Dietary Diversity Score			P (T1 vs T2)	P (T1 vs T3)	P (T2 vs T3)	P^b^
		`1st [< 5.5] (***n*** = 166)	2nd [5.5–6.4] (***n*** = 146)	3rd [> 6.4] (***n*** = 144)				
**Mean DDS score**		5.01	6.05	6.88	–	–	–	–
**Age (years)**		14.01 ± 2.15	14.86 ± 1.96	14.45 ± 2.11	0.004	0.279	0.222	0.005
**Weight (kg)**		49.47 ± 12.19	55.70 ± 14.64	54.93 ± 2.11	< 0.001	0.002	0.890	< 0.001
**Height (cm)**		156.75 ± 9.91	160.22 ± 10.56	159.34 ± 11.68	0.013	0.086	0.765	0.012
**BMI (kg/m2)**		19.88 ± 3.61	21.60 ± 4.35	21.33 ± 4.54	0.001	0.007	0.839	< 0.001
**Waist circumference (cm)**		72.00 ± 8.53	75.81 ± 10.41	75.34 ± 11.62	0.003	0.012	0.919	0.002
**Hip circumference (cm)**		88.50 ± 9.58	90.95 ± 10.29	90.78 ± 10.6	0.085	0.120	0.989	0.057
**Sex [n (%)]**	**Girls**	107 (64.5)	84 (57.5)	76 (52.8)	0.430	0.094	0.689	0.11
	**Boys**	59 (35.5)	62 (42.5)	68 (47.2)				
**Physical activity [n (%)]**	**Low**	53 (31.9)	54 (37)	56 (38.9)	0.312	0.168	0.501	0.41
	**Medium**	113 (68.1)	92 (63)	88 (61.1)				
	**High**	None	None	None				

^a^ Values are presented as Mean ± SD or frequency (percentage)

^b^ Obtained from ANOVA for continuous variables and χ2 test for categorical variables

Age, sex- and energy-adjusted means for dietary intakes across DDS tertile categories are presented in Table 2. Participants in the upper category consumed more vegetables, dairy, energy, protein and calcium, while also consuming less refined grains and carbohydrates. There was no significant association between other food and nutrient intakes of those in the higher and lower tertiles of DDS.

**Table 2:** Dietary intakes of children aged 11–18 years by DDS tertiles categories ^a^

Variables		Tertiles of DDS			P (T1 vs T2) ^**3**^	P (T1 vs T3) ^**3**^	P (T2 vs T3) ^**3**^	P ^**1**^	P ^**2**^
		1st (***n*** = 166)	2nd (***n*** = 146)	3rd (***n*** = 144)					
**Food groups**	**Fruit (gr/d)**	219.75 ± 10.55	218.16 ± 10.92	238.87 ± 8.63	0.993	0.374	0.338	0.28	0.61
	**Vegetables (gr/d)**	153.04 ± 6.25	178.38 ± 7.31	199.70 ± 8.35	0.035	< 0.001	0.107	< 0.001	0.006
	**Meat (gr/d)**	33.50 ± 1.33	42.45 ± 1.99	43.11 ± 1.76	0.001	< 0.001	0.961	< 0.001	0.13
	**Whole grains (gr/d)**	22.83 ± 2.24	26.65 ± 2.38	21.34 ± 1.85	0.426	0.878	0.216	0.22	0.16
	**Refined grains (gr/d)**	323.61 ± 9.07	351.18 ± 11.59	349.61 ± 9.80	0.128	0.162	0.994	0.08	0.03
	**Dairy (gr/d)**	297.49 ± 12.48	351.74 ± 12.83	415.51 ± 11.06	0.005	< 0.001	0.001	< 0.001	< 0.001
	**Fast foods (gr/d)**	11.70 ± 0.95	16.79 ± 1.03	15.90 ± 1.04	0.001	0.009	0.815	0.001	0.10
**Nutrients**	**Energy (kcal)**	1553.40 ± 21.49	1726.55 ± 22.88	1794.02 ± 26.47	< 0.001	< 0.001	0.119	< 0.001	< 0.001
	**Carbohydrate (gr/d)**	61.35 ± 0.35	60.02 ± 0.36	59.55 ± 0.37	< 0.001	< 0.001	0.401	< 0.001	0.002
	**Protein (gr/d)**	13.24 ± 0.12	13.98 ± 0.13	14.43 ± 0.13	< 0.001	< 0.001	0.005	< 0.001	< 0.001
	**Fat (gr/d)**	27.48 ± 0.35	27.89 ± 0.36	28.07 ± 0.37	< 0.001	< 0.001	0.117	< 0.001	0.50
	**Total dietary fiber (gr/d)**	10.63 ± 0.25	11.54 ± 0.24	12.43 ± 0.29	0.044	< 0.001	0.046	< 0.001	0.24
	**Ca (mg/d)**	763.64 ± 18.53	886.88 ± 17.7	985.84 ± 15.28	< 0.001	< 0.001	< 0.001	< 0.001	< 0.001
	**Na (mg/d)**	2511.76 ± 83.16	2701.62 ± 92.83	2901.63 ± 104.58	0.313	0.008	0.300	0.01	0.69

^a^ Values are presented as mean ± SE

^1^ P-value obtained from ANOVA (unadjusted model)

^2^ *P*-value obtained from ANCOVA (Nutrients and food intakes have been adjusted for age and sex for energy intake and age, sex and energy intake for other variables.)

^3^ Between tertile *P*-value Obtained from ANOVA (Tukey method)

The multivariate-adjusted OR and 95% CI of overweight, obesity and abdominal obesity across DDS tertile categories are presented in Table 3. In the crude model, there was a direct association between DDS and overweight (OR 2.13 95% CI 1.26–3.61), obesity (OR 3.45 95% CI 1.41–8.47) and abdominal obesity (OR 3.45 95% CI 1.41–8.47). After adjustment for age and sex, all associations were still significant. Further adjustments for physical activity likewise had no impact on the findings. However, after controlling for energy intake, the associations no longer remained significant for overweight (OR 0.93 95% CI 0.48–1.82), obesity (OR 1.75 95% CI 0.57–5.35) and abdominal obesity (OR 1.68 95% CI 0.55–5.14). The result for abdominal obesity did not change after adjustment for BMI.

**Table 3:** Multivariate-adjusted OR and 95% CI for having abdominal adiposity, overweight and obesity across tertiles of DDS

Variables	Analysis model	1st (***n*** = 166)	2nd (***n*** = 146)	3rd (***n*** = 144)	***P*** trend
		OR	OR (95% CI)	OR (95% CI)	
**Over weight**	Crude	1	1.89 (1.11–3.32)	2.13 (1.26–3.61)	0.005
	Model I	1	2.15 (1.25–3.72)	2.29 (1.34–3.93)	0.003
	Model II	1	2.14 (1.21–3.78)	2.18 (1.24–3.82)	0.007
	Model III	1	1.38 (0.72–2.63)	0.93 (0.48–1.82)	0.8
**Obesity**	Crude	1	2.6 (1.03–6.57)	3.45 (1.41–8.47)	0.007
	Model I	1	2.91 (1.13–7.48)	3.58 (1.44–8.88)	0.006
	Model II	1	2.77 (1.02–7.56)	3.26 (1.24–8.40)	0.02
	Model III	1	2.09 (0.66–6.58)	1.75 (0.57–5.35)	0.41
**Abdominal obesity**	Crude	1	2.22 (0.86–5.73)	3.45 (1.41–8.47)	0.006
	Model I	1	2.35 (0.9–6.14)	3.51 (1.42–8.68)	0.006
	Model II	1	2.20 (0.78–6.19)	3.26 (1.21–8.75)	0.02
	Model III	1	1.46 (0.45–4.73)	1.68 (0.55–5.14)	0.38
	Model IV	1	0.66 (0.11–3.98)	2.16 (0.38–8.32)	0.24

Overweight and obesity were defined based on 85th ≤ BMI <95th and ≥ 95th percentiles of BMI, respectively. Also abdominal obesity was considered as WC ≥ 85th percentiles

Model I: adjusted for age and sex

Model II: further adjusted for physical activity

Model III: more adjustment for total energy intake

Model IV: additionally adjusted for BMI

Table 4 shows the multivariate-adjusted OR and 95% CI of overweight, obesity and abdominal obesity across DDS tertile categories for grains, vegetables, fruits, dairy and meat. As can be seen from Table 4, a higher intake of grains is associated with an increased risk of overweight in both the crude (OR 5.11 95% CI 2.90–6.00) and the adjusted model (OR 2.72 95% CI 1.28–5.77). A significant association between grains variety intake and obesity and abdominal obesity was observed in the crude model (OR 3.74 95% CI 1.63–8.60 for obesity and OR 4.18 95% CI 1.77–9.85 for abdominal obesity), but not the adjusted model. Those in the highest tertile of diversity score for vegetables compared to the lowest tertile were at a 65% decreased risk of overweight in the adjusted model (95% CI 0.12–1.00). Obesity and abdominal obesity were not associated with intake of a variety of vegetables in either the crude or the adjusted model. In the fruit group, participants in the highest tertile of intake of a variety of fruits showed a 53% lower risk of overweight (95% CI 0.22–0.10) and an 81% decreased risk of obesity (95% CI 0.05–0.74). Additionally, intake of a higher variety of fruits was associated with a decreased risk of abdominal obesity in the crude model (OR 0.32 95%CI 0.12–0.82) but after adjustment for cofounder variables, the association was no longer significant. More varied dairy intake was not associated with overweight, obesity and abdominal obesity in both crude and adjusted models. A higher variety of meat intake was also positively associated with overweight in the crude model (OR 4.24 95% CI 1.38–6.98) but in the adjusted model there was no association between a variety of meat intake and overweight, obesity and abdominal obesity.

**Table 4:** Multivariate odds ratio (95% confidence interval) of overweight, obesity and abdominal obesity by tertile of the diversity score for grains, vegetables, fruit, dairy and meats

Variables		Analysis models	Tertile of Dietary Diversity Score		
			1st (***n*** = 166)	2nd (***n*** = 146)	3rd (***n*** = 144)
**Grains**	**Overweight**	Crude	1	2.45 (1.45–4.13)	5.11 (2.90–6.00)
		Multivariable-adjusted ^a^	1	1.58 (0.81–3.07)	2.72 (1.28–5.77)
	**Obesity**	Crude	1	1.90 (0.83–4.34)	3.74 (1.63–8.60)
		Multivariable-adjusted ^a^	1	0.78 (0.25–2.42)	0.93 (0.28–3.07)
	**Abdominal obesity**	Crude	1	1.96 (0.83–4.66)	4.18 (1.77–9.85)
		Multivariable-adjusted ^a^	1	0.74 (0.11–4.90)	1.37 (0.23–8.20)
**Vegetables**	**Overweight**	Crude	1	1.39 (0.77–2.48)	1.54 (0.76–3.11)
		Multivariable-adjusted ^a^	1	1.03 (0.46–2.31)	0.35 (0.12–1.00)
	**Obesity**	Crude	1	0.94 (0.39–2.27)	1.91 (0.71–5.11)
		Multivariable-adjusted ^a^	1	0.70 (0.18–2.71)	0.58 (0.13–2.67)
	**Abdominal obesity**	Crude	1	1.16 (0.46–2.95)	1.83 (0.63–5.28)
		Multivariable-adjusted ^a^	1	3.28 (0.28–8.86)	1.16 (0.07–8.02)
**Fruits**	**Overweight**	Crude	1	0.96 (0.58–1.58)	0.70 (0.42–1.77)
		Multivariable-adjusted ^a^	1	1.11 (0.56–2.20)	0.47 (0.22–0.10)
	**Obesity**	Crude	1	0.87 (0.43–1.78)	0.35 (0.14–0.86)
		Multivariable-adjusted ^a^	1	1.08 (0.39–3.01)	0.19 (0.05–0.74)
	**Abdominal obesity**	Crude	1	0.93 (0.45–1.91)	0.32 (0.12–0.82)
		Multivariable-adjusted ^a^	1	1.74 (0.63–2.27)	1.20 (0.16–4.05)
**Dairy**	**Overweight**	Crude	1	1.53 (0.68–2.79)	0.97 (0.89–3.01)
		Multivariable-adjusted ^a^	1	2.15 (0.92–4.57)	0.89 (0.39–2.01)
	**Obesity**	Crude	1	3.21 (0.23–4.56)	1.14 (0.52–3.18)
		Multivariable-adjusted ^a^	1	5.04 (0.68–5.58)	2.98 (0.62–6.42)
	**Abdominal obesity**	Crude	1	2.47 (0.73–5.91)	2.94 (0.63–4.23)
		Multivariable-adjusted ^a^	1	3.27 (0.24–5.46)	4.75 (0.47–7.61)
**Meats**	**Overweight**	Crude	1	3.49 (1.21–6.07)	4.24 (1.38–6.98)
		Multivariable-adjusted ^a^	1	3.25 (0.82–6.86)	3.23 (0.74–7.13)
	**Obesity**	Crude	1	4.40 (0.59–6.09)	4.34 (0.53–7.43)
		Multivariable-adjusted ^a^	1	3.02 (0.30–6.10)	1.98 (0.16–4.15)
	**Abdominal obesity**	Crude	1	4.40 (0.59–5.09)	3.30 (0.39–7.70)
		Multivariable-adjusted ^a^	1	0.63 (0.01–3.93)	0.23 (0.00–1.94)

^a^Adjusted for age, sex, energy intake, physical activity, mutual effects of other groups for each group and BMI for abdominal obesity

## Discussion

This study was conducted on a representative sample of Iranian school-aged children, and the results showed a positive association between DDS and obesity via higher energy intake. This population was chosen due to the comparatively little nutritional information for the specific age group in Iran. As DDS reflects an aggregate view of the diet, it is a useful indicator to assess the correlations between diet and diseases.

Few observational studies have directly assessed the association between DDS and childhood obesity, suggesting that there are some inconsistent reports in this regard. The results of the present study showed a direct association between DDS and energy intake, which are in line with those of previous studies showing a positive association between DDS and energy intake [25, 26]. In a study conducted in Mali [27] and another study on adults in Tehran [28], a positive correlation was found between DDS and energy intake. According to these results, energy is an important component of increased food variety, and can be a limitation for a variety of indices in health promotion programs. Therefore, the consumption of a varied diet while staying within energy needs is recommended [29].

In the present study, an increased dietary diversity score is related to increasing energy intake of vegetables. However, in other obesity-protective groups such as fruits and whole grains, no difference was observed in DDS tertiles. In this study, the diversity score of vegetables and fruit groups was inversely associated with overweight and obesity, thus these results can explain the protective effects of a varied diet on obesity through increased consumption of fruits and vegetables.

The results from this study are in contrast to results of previous studies showing an inverse association between DDS and obesity. In a study conducted by Azadbakhet et al. * [16] on Iranian adults, a negative correlation was observed between DDS, obesity and abdominal obesity. In addition, two studies conducted by Kant et al. * [30, 31] showed an inverse relationship between DDS and BMI. In contrast, in a study of US preschool children * [32], a higher dietary diversity score was positively associated with BMI. Also, in a study by Jayawardena et al. [17], a higher diversity score was associated with obesity in adults. Overall, a meta-analysis failed to show an association between dietary diversity and obesity [1].

As stated in previous studies, these inconsistent findings may be due to different serving cutoff points used to describe the dietary diversity. For example, consumption of at least half a serving of each food category in the study of Mirmiran et al. [33] and our study, or consumption of at least 10 g of selected food items in Kennedy et al. study [34] and awarding a point to any food item consumed daily or in the previous week in the study of Chua et al. [35] are of some criterions to count toward the diversity score in different studies.

However, the diversity score calculated in this study has two advantages over other studies. The first is that, while most previous studies had used dietary recalls to evaluate DDS, here it was achieved using FFQ, which is a more applicable instrument as dietary diversity refers to long dietary exposure. The second point is that DDS calculations were derived from 23 subgroups of five main food groups. This resulted in higher DDS accuracy, as it represents the diversity of food groups as well as the diversity within food groups.

Despite the association of obesity with a more diverse diet in the present study, after adjustment for energy consumption, the risk of obesity was decreased in the highest DDS category, but it was not statistically significant. It is therefore better to suggest increasing the diversity score with fruits, vegetables and whole grains which have favorable effects on obesity.

The strengths of this study include the large sample size, the school-aged population and the consideration of potential confounders controlled for in the reported models of the logistic regression. There are also some limitations to this study. Due to the cross-sectional study design, a prospective association remains to be identified. Because of the FFQ usage, the misclassification of biases should be noted. Although efforts were made to control for known confounders, residual confounding cannot be excluded in these findings. The prevalence of obesity was low among the study populations. It could perhaps be better to analyze overweight and obese individuals together in one model, rather than in separate models. In addition, because the study was conducted only in Isfahan, it is difficult to generalize the results of the study to the wider adolescent population of Iran.

## Conclusion

In conclusion, this study suggests that dietary diversity score is positively associated with obesity and abdominal obesity; however, this association is not independent of energy intake. Therefore, despite the importance of dietary diversity as an indicator of nutrient adequacy, increasing the variety of foods in children’s diets requires the careful consideration in terms of energy intake, and dietary diversity should be improved in selective food items. Also, more well-designed prospective studies are needed to clarify the effect of dietary diversity on childhood obesity.

## Data Availability

The datasets generated and/or analysed during the current study are not publicly available as per the rules and regulations of the Clinical Nutrition department of Isfahan University of Medical Science but are available from the corresponding author on reasonable request.

## Abbreviations

DDS: Dietary Diversity Score
FFQ: Food frequency questionnaire
WC: Waist circumference
PAQ: Physical Activity Questionnaire
ANCOVA: Analysis of covariance
OR: Odd Ratio
SD: Standard Deviation
CI: Confidence Interval
